# Association between lone star tick bites and increased alpha-gal sensitization: evidence from a prospective cohort of outdoor workers

**DOI:** 10.1186/s13071-020-04343-4

**Published:** 2020-09-14

**Authors:** Cedar L. Mitchell, Feng-Chang Lin, Meagan Vaughn, Charles S. Apperson, Steven R. Meshnick, Scott P. Commins

**Affiliations:** 1 Department of Epidemiology, Gillings School of Global Public Health, University of North Carolina, Chapel Hill, North Carolina USA; 2 Department of Biostatistics, Gillings School of Global Public Health, University of North Carolina at Chapel Hill, Chapel Hill, North Carolina USA; 3 Department of Entomology and Plant Pathology, North Carolina State University, Raleigh, North Carolina USA; 4 Department of Medicine, Thurston Research Center, Division of Allergy, Immunology and Rheumatology, Chapel Hill, North Carolina USA

**Keywords:** Alpha-gal, *Amblyomma americanum*, Red meat allergy

## Abstract

**Background:**

Alpha-gal is an oligosaccharide implicated in delayed anaphylaxis following red meat consumption. Exposure to tick bites has been correlated with development of an allergic response to alpha-gal. However, evidence prospectively linking exposure to a single tick species and an immune response to alpha-gal is lacking.

**Methods:**

We used serum samples from a prior study cohort of outdoor workers in North Carolina, USA, with high exposure to the lone star tick, *Amblyomma americanum*, to prospectively evaluate the relationship between tick bites and anti-alpha-gal IgE antibodies.

**Results:**

Individuals who reported exposure to one or more tick bites were significantly more likely to have a positive change in anti-alpha-gal IgE compared to individuals with no reported tick bites. This relationship was not dependent on time. A trend toward increasing number of tick bites and increased anti-alpha-gal IgE levels was observed but not statistically significant.

**Conclusion:**

To our knowledge, this is the first study to prospectively link documented exposure to *A. americanum* bites and increased sensitization to alpha-gal in a cohort of outdoor workers. Our results support the role of *A. americanum* as likely agents for eliciting an allergic response to red meat, and highlight the importance of preventing tick bites.
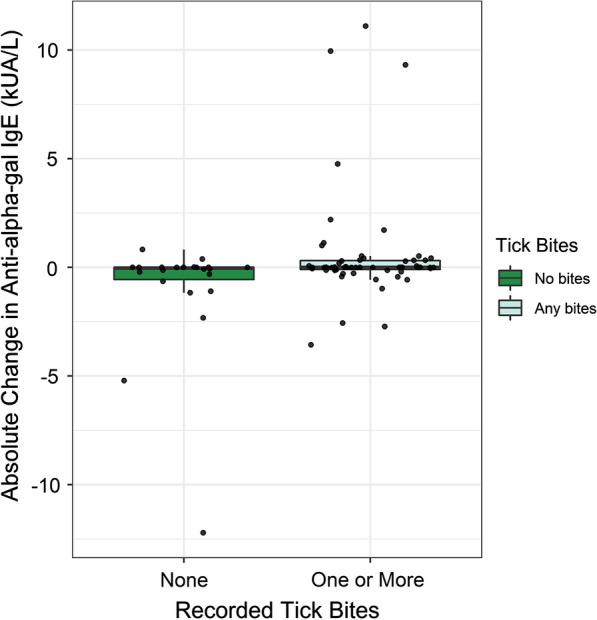

## Background

Alpha-gal syndrome (AGS) is an immunoglobulin E (IgE)-mediated allergy to the oligosaccharide galactose-alpha-1,3-galactose (alpha-gal), present in non-primate mammals [1]. AGS is characterized by a delayed onset allergic reaction following ingestion of mammalian meat (e.g. beef, pork, lamb) or its derivatives, and a positive serum IgE result to alpha-gal (≥ 0.1 kU/l) [1, 2] Since 2009, AGS has been widely reported in North America, Australia, Europe and Asia, with bites from various tick species often suggested as a proximate cause of AGS [3–6]. While these previous reports have proposed a relationship between tick bites and AGS, the nature of the study designs (case reports [1, 3], cross-sectional studies [4, 5] or retrospective epidemiological studies [6]) preclude recording of the exposure prior to the outcome and are vulnerable to recall bias and exposure misclassification. Thus, evidence from prospective studies is needed to confirm the role of tick bites in the development of AGS.

In the USA, most cases of AGS have been reported from the Southeast, where the lone star tick, *Amblyomma americanum*, is a prevalent and problematic human-biting species [7, 8]. Lone star tick bites have been retrospectively or circumstantially linked to the development of AGS [1, 3]; however, to date no study has evaluated changes in immune sensitization to alpha-gal following documented exposure to *A. americanum* bites. The purpose of this study was to prospectively define the relationship between *A. americanum* tick bites and anti-alpha-gal IgE antibody levels in a cohort of outdoor workers in the state of North Carolina, USA.

## Methods

### Study design and participants

This study analyzed serum samples from a previous randomized control trial (RCT) of North Carolina outdoor workers [9]. Briefly, the RCT enrolled North Carolina state park and forestry employees who worked outdoors ≥ 10 hours/week during peak tick season, were over age 18, were not pregnant or planning to become pregnant during the study, had no known sensitivities to insecticides, and provided written informed consent. The study spanned two tick exposure seasons defined as March to October of 2011 and 2012 and was conducted to evaluate the protective effect of permethrin treated clothing for the prevention of tick bites [9]. Serum samples were collected before and after each tick exposure season during the initial RCT to test for the presence of tick-borne pathogens. Samples were only included in this study for participants who provided written informed consent for specimen storage and use for consecutive studies during the RCT. Inclusion of samples was further restricted to participants with paired sera that spanned at least one tick exposure season and were collected ≤ 12 months apart. The use of paired sera allowed for the evaluation of the change in alpha-gal at the individual level to control for variation in baseline alpha-gal titers across participants.

### Anti-alpha-gal IgE antibody measurement and tick exposure assessment

Levels of anti-alpha-gal IgE were measured for all included samples using a previously validated ImmunoCAP assay [1]. A threshold for anti-alpha-gal IgE response of > 0.1 kUA/l was used to maximize sensitivity to antibody levels which have been detected as low as 0.1kUA/l among patients with clinical symptoms and allergic reactions to alpha-gal [2]. Tick bites were recorded using weekly logs during the parent study and removed ticks were sent by study participants to an entomology laboratory at North Carolina State University for identification. Baseline participant characteristics and weekly tick bite exposure data were extracted from the parent study.

### Statistical analysis

The main exposure variable was tick bites, dichotomized as ≥ 1 bites or 0 bites recorded between serum collection times. The outcome was measured as an absolute change in anti-alpha-gal IgE levels between serum collections. A linear mixed-effects model assuming compound symmetry correlation across time was used to evaluate the change in anti-alpha-gal IgE levels in response to tick exposure (any bites versus none) and time as the predictors. The analysis was conducted using all paired sera pooled across study years, and included a random intercept to control for repeated measures. A threshold of 0.05 was used for statistical significance testing.

## Results and discussion

### Characteristics of the study population

There were 81 paired samples from 52 study participants meeting eligibility requirements. The average age of participants was 40 years (range: 25–58 years), 79% were male; 73% (59/81) of paired samples had ≥ 1 tick bite during the study period. A total of 358 ticks were sent in by 41 participants for identification across the study period. Of these, 97% (347/358) were identified as *A. americanum* of which, 58% were nymphs or larvae. The other 3% of submitted ticks were identified as *Dermacentor variabilis* (*n* = 7), *Amblyomma maculatum* (*n* = 3) and *Ixodes scapularis* (*n* = 1). All participants who reported one or more tick bites and sent in ticks for identification had documented exposure to 1 or more *A. americanum* ticks. Of the 11 participants who did not send in ticks, 8 reported no tick bites during the study period.

The prevalence of anti-alpha-gal IgE antibodies > 0.1kUA/l among participants at baseline, regardless of tick exposure, was 58% (30/52), at the end of the first year it was 60% (31/52), and among those who completed two years of the study, it was 69% (20/29). Of the participants with undetected anti-alpha-gal antibodies at baseline, new sensitizations appeared in 4 individuals within their first year of the study. The overall median anti-alpha-gal IgE levels were 0.29, 0.22, and 0.25 kUA/l at baseline, the end of study year 1, and the end of study year 2, respectively.

### Association between tick bites and alpha-gal sensitization

**Fig. 1:**
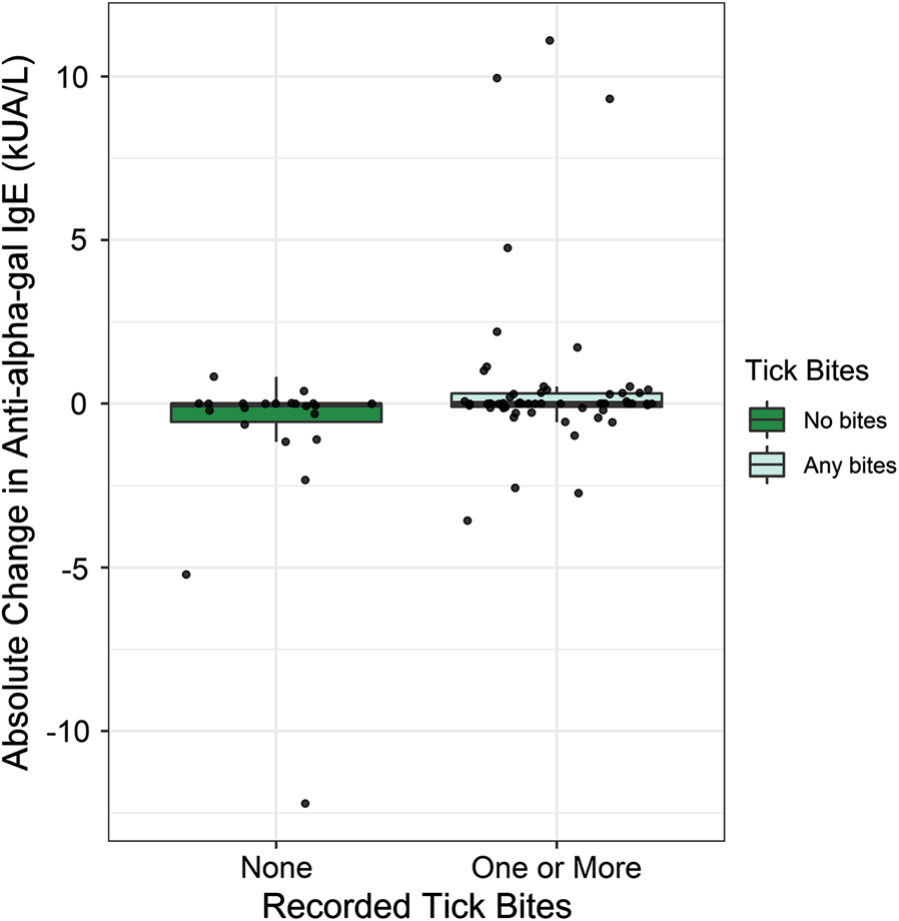
Distribution of absolute change in alpha-gal IgE sensitization across paired samples, grouped by tick bite exposure represented by boxplots. For each group, the box is bound by the upper and lower quartile of data as distributed about the median, vertical lines extend from the box to the maximum and minimum ranges of the data estimated by 1.5*interquartile range. Data points representing the absolute change in alpha-gal IgE for each sample overlay the boxplots. *Abbreviations:* IgE, immunoglobulin E

Results from our mixed effects analysis revealed that exposure to one or more tick bites was significantly associated with an increase in mean anti-alpha-gal IgE (estimate = 1.99, 95% CI: 0.39–3.60, *P* = 0.02). Participants who reported any tick bites experienced an average increase in alpha-gal sensitization of 1.99 kUA/l (95% CI: 0.39–3.60 kUA/l) compared to individuals who reported no tick bites, controlling for time and individuals with repeated measures. The relationship between tick bites and change in alpha-gal sensitization did not significantly depend on study year (estimate = 0.25, 95% CI: − 2.10–2.60, *P* = 0.84). The distribution of changes in anti-alpha-gal levels among participants based on their tick bite exposure status during both study years is shown in Fig. 1.

### Secondary dose response analysis

**Fig. 2:**
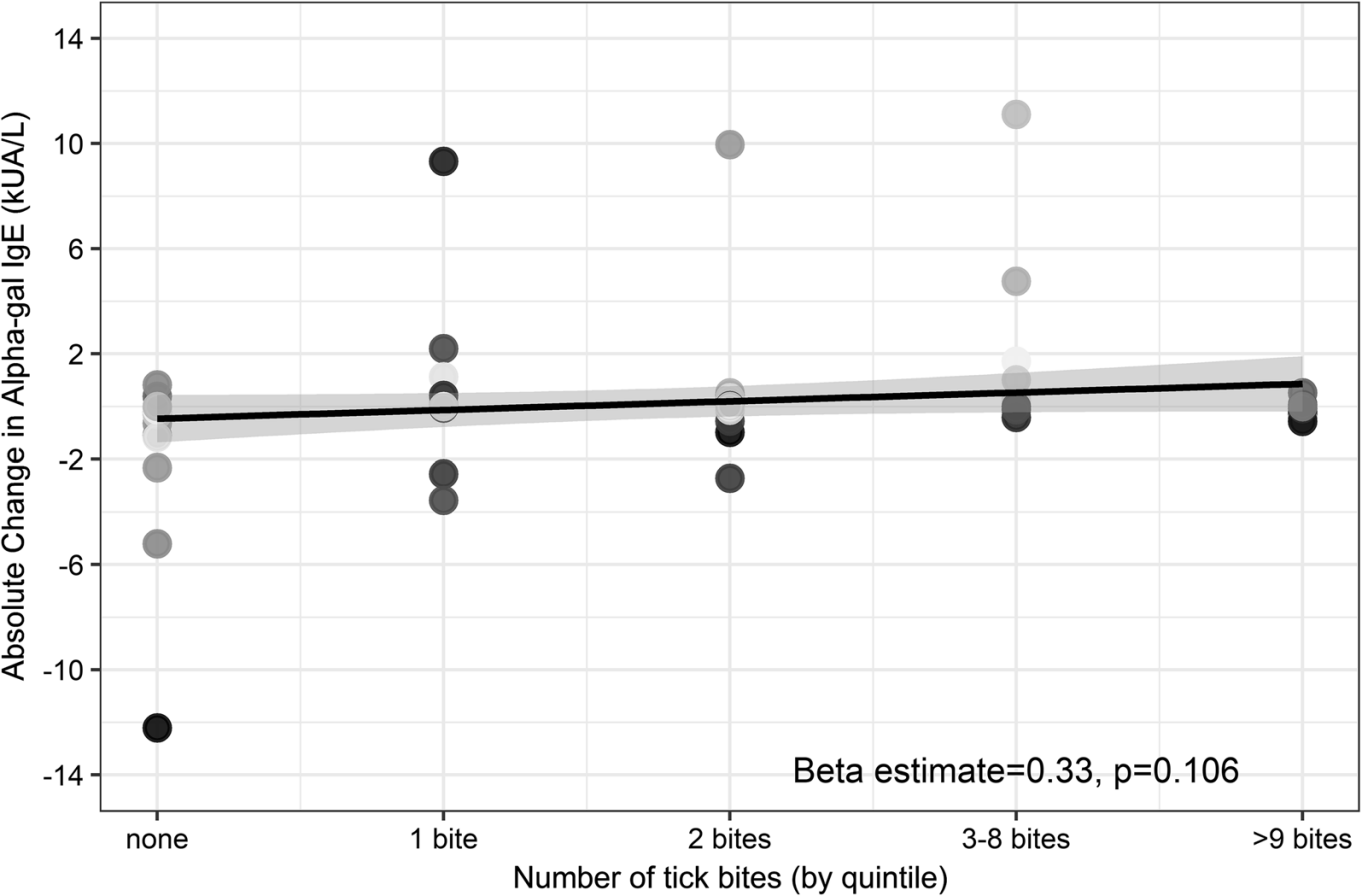
Change in alpha-gal IgE sensitization tends to increase with the number of reported tick bites; however, the estimated average increase of 0.33 kUA/L of alpha-gal IgE for each 1-quintile increase in reported tick bites is not significant (estimate: 0.33, CI: − 0.07 to 0.73, *P* = 0.106). *Abbreviations:* IgE, immunoglobulin E; CI, confidence interval

We also investigated whether differences in anti-alpha-gal IgE levels were associated with the number of tick bites. Tick bite numbers were binned by quintiles of occurrence across the full study. Quintile intervals were zero bites, 1 bite, 2 bites, 3–8 bites, and ≥ 9 bites. A tendency toward increased change in anti-alpha-gal IgE with increased tick exposure was observed (Fig. 2), but it was not statistically significant (estimate = 0.33, 95% CI: − 0.07–0.73, *P* = 0.106). This observed pattern does not change among participants with a baseline sensitivity to alpha-gal (anti-alpha-gal IgE > 0.1kUA/l), data not shown.

### Study limitations

The major study limitation is the small sample size and lack of information regarding symptoms commonly associated with AGS. As a result, there were no reported cases of red meat allergy. Although the findings are limited by exposure to one tick species, *A. americanum*, appears to be the most relevant species in the USA based on geographical range [8]. We also note the high baseline sensitivity of this cohort to anti-alpha-gal antibodies (58% positive at baseline) presumably due to exposure to *A. americanum* prior to enrollment in the parent study. This may have reduced the effect size observed in this study as opposed to a sample of *A. americanum* naïve individuals. Acknowledging these limitations, this is the first report to prospectively link *A. americanum* bites to significantly increased anti-alpha-gal IgE.

## Conclusions

Our results show that documented exposure to at least one *A. americanum* bite is associated with statistically significant positive change in anti-alpha-gal IgE levels. While our study found that any exposure to *A. americanum* bites is significantly associated with increased sensitization to alpha-gal, we were unable to detect a significant dose response between the number of tick bites and increasing anti-alpha-gal IgE levels, likely due to the small study size. Additional studies are needed to fully characterize the relationship between the frequency of tick bites and changes in alpha-gal sensitization. However, the results presented here indicate that anti-alpha-gal IgE antibody levels increase following documented exposure to the lone star tick and support the importance of preventing tick bites as a measure of protection against increased alpha-gal sensitization.

## Data Availability

The datasets used for analysis during this study are available from the corresponding author upon request.

## Abbreviations

AGS: alpha-gal syndrome
IgE: immunoglobulin E
RCT: randomized control trial
